# Euglycemic Ketoacidosis in Spinal Muscular Atrophy

**DOI:** 10.1155/2019/2862916

**Published:** 2019-01-27

**Authors:** Dimitrios Stoimenis, Christina Spyridonidou, Sofia Theofanidou, Nikolaos Petridis, Nikos Papaioannou, Christina Iasonidou, Nikolaos Kapravelos

**Affiliations:** ^1^Department of Internal Medicine, General Hospital G. Papanikolaou, Thessaloniki, Greece; ^2^Intensive Care Unit, General Hospital Papageorgiou, Thessaloniki, Greece; ^3^Department of Internal Medicine, General Hospital G. Gennimatas, Athens, Greece; ^4^Intensive Care Unit, General Hospital G. Papanikolaou, Thessaloniki, Greece

## Abstract

Euglycemic ketoacidosis is defined by the triad of high anion gap acidosis, increased plasma ketones, and the absence of hyperglycemia. Apart from diabetes mellitus, the disorder may occur in prolonged fasting, excessive alcohol consumption, pregnancy, and inborn errors of metabolism. Here, we highlight the diagnosis of euglycemic ketoacidosis in a pediatric nondiabetic patient with spinal muscular atrophy (SMA) type 1 (Werdnig–Hoffmann disease), who, subsequently to her postoperative admission to the intensive care unit following a spinal surgery, developed high anion gap metabolic acidosis. We discuss the pathophysiology of acid-base disorders in SMA, along with the glucose and fatty acids metabolism, the necessary knowledge for medical practitioners.

## 1. Introduction

Euglycemic ketoacidosis, albeit underrated, has been acknowledged since 1973, most commonly occurring in patients with diabetes mellitus (DM) [1]. The American Diabetes Association suggested the definition of euglycemic diabetic ketoacidosis as blood glucose <13.9 mmol/L combined with ketoacidosis [2]. This disorder, however, can develop in the absence of DM, under conditions characterized by severe ketosis, namely, starvation, alcohol intoxication, pregnancy, and inborn errors of metabolism [2]. Herein, we describe the diagnosis of euglycemic ketoacidosis in a pediatric nondiabetic patient with a known history of spinal muscular atrophy (SMA).

## 2. Case Presentation

**Figure 1:**
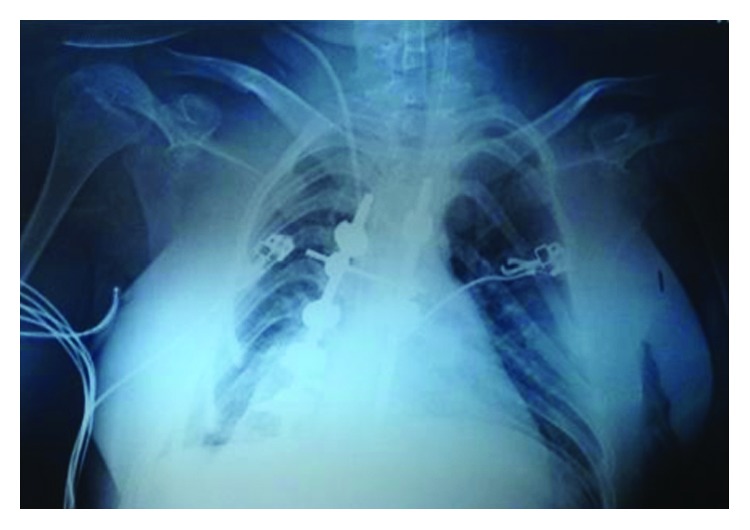
The spinal malformation and the spondylodesis materials of the pediatric patient.

A 13-year-old female patient was postoperatively admitted to the intensive care unit (ICU), following a spondylodesis procedure due to severe spinal malformation (Figure 1). The girl's medical history was remarkable for a genetically confirmed diagnosis of SMA type 1 (Werdnig–Hoffmann disease) within her first six months of age (homozygous deletion of the survival motor neuron 1 (SMN1) on exon 7, 5q chromosome, with two copies of the SMN2 gene).

On the third ICU day, the patient developed metabolic acidosis. Arterial blood gases revealed pH 7.17 (reference 7.35–7.45), partial pressure of oxygen (PaO_2_) 12.40 kPa (reference 11–13 kPa), partial pressure of carbon dioxide (PaCO_2_) 4 kPa (reference 4.7–6.0 kPa), bicarbonate (HCO_3_^−^) 10.7 mmol/L (reference 22–26 mmol/L), and base deficit −13 mmol/L. Lactate was normal with a value of 0.8 mmol/L (reference 0.56–2.0 mmol/L). The anion gap was 14 mmol/L, and the corrected value for the albumin anion gap was 26 mmol/L (reference 3–11 mmol/L).

Before admission, the patient's respiratory function was impaired. She had significant respiratory muscle weakness and poor cough ability, and she required at home the use of noninvasive ventilation (NIV) and mechanically assisted coughing (MAC). She was intubated prior to surgery and extubated after a difficult and prolonged weaning following admission to the ICU. At the time of examination, she was breathing spontaneously and was supported intermittently with NIV. Despite her having had a gastrostomy tube at an earlier stage of life, during hospitalization, she was fasted for three consecutive days, the day of the surgery and the next two postoperative days, in light of the presumed risk of pulmonary aspiration. Furthermore, she was apyrexial, intravenously hydrated with 0.9% normal saline, and not on any inotropic or vasopressor support since her vital signs implied hemodynamic stability (systolic blood pressure > 110 mmHg and heart rate < 90 bpm). Common causes of metabolic acidosis with a high anion gap, namely, uremia, diabetic or alcoholic ketoacidosis, and lactic acidosis, were excluded. Renal and liver functions were normal, and no sign of infection was evident since inflammatory markers were negative and white blood cell account was normal. Total parenteral nutrition, and other agents, such as valproic acid and salicylates, which can potentially induce acidosis, had not been administered.

The pediatric patient had no history of DM, and she presented normal glycated hemoglobin (4.4%, reference 4.0–5.7%) and plasma glucose levels. However, her reduced body mass index-for-age (14.8 kg/m^2^) and, accordingly, the extremely low levels of serum urea (1.78 mmol/L, reference 2.5–8.0 mmol/L) and serum creatinine (8.8 mmol/L, reference 52–96.8 mmol/L) prompted the ICU consultation team to investigate the likelihood of ketosis and ketoacidosis. Plasma *β*-hydroxybutyrate assay was unavailable on the premises; nevertheless, urine analysis revealed a severe degree of ketonuria (acetoacetate >7.84 mmol/L; urine value 4+).

Upon establishment of the diagnosis of euglycemic ketoacidosis, our patient was started on a fat-free enteral nutrition enriched with carbohydrates and proteins via her gastrostomy feeding tube. The acidosis resolved completely within 48 hours; urine ketones were negative at the time. No bicarbonate or insulin infusion was administered. The patient was discharged to the ward four days later.

## 3. Discussion

SMA comprises an autosomal recessive neuromuscular disorder and the most frequent inherited motor neuron disease, characterized by insufficient produced levels of the survival motor neuron protein, which results in progressive neurodegeneration and atrophy of the skeletal muscles [3]. In patients with SMA, spinal deformity, respiratory complications, and orthopedic disorders constitute the major challenges of their clinical management. SMA is classified into four types, with type 1 representing the most severe form of the disease (Werdnig–Hoffmann disease) with infantile onset and multiorgan impairment [3]. Of note, due to the severity of the clinical course in SMA type 1, the majority of patients with this phenotype do not achieve the spinal developmental milestone that was described in our case, which was rather an atypically mild presentation. Suggestively, in a previous reported retrospect chart review and caregiver questionnaire for referred children with SMA type 1, only four patients with atypically mild SMA type 1 underwent a surgical correction of scoliosis, while none of the 74 typical SMA type 1 patients achieved this degree of spinal development in order to require such a surgical correction [4].

Additionally, SMA is associated with a complex disorder of fatty acids metabolism, which has raised controversy over the exact underlying pathogenesis. While it has been postulated that SMA patients are particularly susceptible to fasting hypoglycemia and ketosis mostly attributed to their reduced muscular mass [5, 6], several authors have indicated a mitochondrial *β*-oxidation abnormality in muscles as the primary pathogenic mechanism of fatty acids metabolism in SMA [3]. In fact, it has been reported that these patients may present increased levels of serum dicarboxylic acids, dicarboxylic aciduria, and potential metabolic acidosis during stress or fasting periods [7, 8].

In the case presented, over the first two postoperative days, the pediatric patient was fasted, given the high risk of pulmonary aspiration. In light of the absence of DM, typical predisposing factors of high anion gap acidosis [9], such as diabetic and alcoholic ketoacidosis, uremia, lactic acidosis, parenteral nutrition, and drug-induced acidosis, were ruled out from the differential diagnosis. Although no further anthropometric study, other than body mass index-for-age, was performed in order to assess the exact baseline nutritional status (e.g., skinfold thickness or dual energy X-ray absorptiometry), we considered that the patient had an element of chronic malnutrition. Hence, the reduced muscular mass combined with the fasting state, and the perioperative stress led the ICU consultation team to presume that the patient could have been subject to severe ketosis due to her proneness to rapid depletion of liver and muscle glycogen stores [10]. In fact, starvation and stress-induced acidosis perioperatively and in the ICU setting have previously been described in certain circumstances [11, 12], in which several factors in combination can result in moderate or severe ketoacidosis. Repeated episodes of stress-induced ketoacidosis were also reported in an adult patient with SMA type 2, and it was suggested that this metabolic disorder can be easily corrected within hours or few days, provided it is recognized early [13]. Of note, in our case, during the surgery and the first two postoperative days in the ICU, our patient was administered only normal saline solutions, and it is likely that should had we hydrated the patient more aggressively with crystalloids and dextrose solutions, she would rather not have developed the metabolic acidosis, at least to this degree.

Regarding our therapeutic approach, the applicable diet alone, a mixture of carbohydrates and proteins, resulted in the complete resolution of ketoacidosis, without applying any other treatment and thus confirmed the initial thought process of clinical diagnosis. The rationale for this fat-free dietary management was the reduction of the patient's further exposure to fatty acid substrates during the metabolic derangement in order to prevent additional accumulation of potentially toxic-free fatty acids [14] and the inhibition of ketosis by promoting glycolysis and glycogenesis, through appropriate enteral nutrition and adequate carbohydrate intake [10]. Certainly, apart from the need for sufficient continuous carbohydrate intake, we do not recommend or imply that a long-term fat-free diet could represent a suitable nutrition in patients with SMA, given that the deprivation of lipids or vital fat-soluble vitamins in the diet would have unknown implications and risks. Furthermore, it is worth noting that we did not assess the patient with tandem mass spectrometry analyses of the acylcarnitine profile and free fatty acids or with urine organic acid investigations for known inborn errors of metabolism, such as organic acidemias and ketolytic defects [2, 8]. Therefore, post hoc, we cannot definitely exclude the presence of other acids, including dicarboxylic acids, which could have contributed in the metabolic acidosis and neither can we determine the exact pathogenic mechanisms of abnormal fatty acids metabolism in our patient.

In conclusion, this case emphasizes the metabolic derangement in a patient with SMA type 1 after short-term fasting in the ICU setting and in a state of perioperative stress. Moreover, our report points out that clinicians and pediatric physicians should not forget the entity of nondiabetic euglycemic ketoacidosis when deciphering high anion gap acidosis. In conjunction with a pertinent medical history and clinical indication, urine ketones testing, a rapid and low-cost assay, can have particular diagnostic value with regard to abnormal glucose and fatty acids metabolism, and therefore, should not be neglected in the absence of DM.

## References

[1] Munro J. F., Campbell I. W., McCuish A. C., Duncan L. J. P. (1973). Euglycaemic diabetic ketoacidosis. *BMJ*.

[2] Le Neveu F., Hywel B., Harvey J. N. (2013). Euglycaemic ketoacidosis in patients with and without diabetes. *Practical Diabetes*.

[3] Shababi M., Lorson C. L., Rudnik-Schöneborn S. S. (2013). Spinal muscular atrophy: a motor neuron disorder or a multi-organ disease?. *Journal of Anatomy*.

[4] Bach J. R. (2007). Medical considerations of long-term survival of Werdnig–Hoffmann disease. *American Journal of Physical Medicine & Rehabilitation*.

[5] Bruce A. K., Jacobsen E., Dossing H., Kondrup J. (1995). Hypoglycemia in spinal muscular atrophy. *The Lancet*.

[6] Lakkis B., El Chediak A., Hashash J. G., Koubar S. H. (2018). Severe ketoacidosis in a patient with spinal muscular atrophy. *CEN Case Reports*.

[7] Kelley R. I., Sladky J. T. (1986). Dicarboxylic aciduria in an infant with spinal muscular atrophy. *Annals of Neurology*.

[8] Crawford T. O., Sladky J. T., Hurko O., Besner-Johnston A., Kelley R. I. (1999). Abnormal fatty acid metabolism in childhood spinal muscular atrophy. *Annals of Neurology*.

[9] Reddy P., Mooradian A. D. (2009). Clinical utility of anion gap in deciphering acid-base disorders. *International Journal of Clinical Practice*.

[10] Laffel L. (1999). Ketone bodies: a review of physiology, pathophysiology and application of monitoring to diabetes. *Diabetes/Metabolism Research and Reviews*.

[11] Toth H. L., Greenbaum L. A. (2003). Severe acidosis caused by starvation and stress. *American Journal of Kidney Diseases*.

[12] Mostert M., Bonavia A. (2016). Starvation ketoacidosis as a cause of unexplained metabolic acidosis in the perioperative period. *American Journal of Case Reports*.

[13] Mulroy E., Gleeson S., Furlong M. J. (2016). Stress-induced ketoacidosis in spinal muscular atrophy: an under-recognized complication. *Journal of Neuromuscular Diseases*.

[14] Tein I., Sloane A. E., Donner E. J., Lehotay D. C., Millington D. S., Kelley R. I. (1995). Fatty acid oxidation abnormalities in childhood-onset spinal muscular atrophy: primary or secondary defect(s)?. *Pediatric Neurology*.

